# Association between resistance to cerebrospinal fluid flow and cardiac-induced brain tissue motion for Chiari malformation type I

**DOI:** 10.1007/s00234-023-03207-9

**Published:** 2023-08-30

**Authors:** Saeed Mohsenian, Alaaddin Ibrahimy, Mohamad Motaz F. Al Samman, John N. Oshinski, Rafeeque A. Bhadelia, Daniel L. Barrow, Philip A. Allen, Rouzbeh Amini, Francis Loth

**Affiliations:** 1 Department of Bioengineering, Northeastern University, 360 Huntington Ave, Boston, MA 02115 USA; 2 Department of Biomedical Engineering, Yale University, 17 Hillhouse Ave, New Haven, CT 06520 USA; 3 Departments of Radiology & Imaging Sciences and Biomedical Engineering, Emory University School of Medicine, 1364 Clifton Road NE, Atlanta, GA 30322 USA; 4 Department of Radiology, Beth Israel Deaconess Medical Center & Harvard University School of Medicine, 330 Brookline Ave, Boston, MA 02215 USA; 5 Department of Neurosurgery, Emory University School of Medicine, 1364 Clifton Road NE, Atlanta, GA 30322 USA; 6 Department of Psychology, The University of Akron, 302 E Buchtel Ave, Akron, OH 44325 USA; 7 Departments of Mechanical and Industrial Engineering, and Bioengineering, Northeastern University, 805 Columbus Ave, ISEC 508, Boston, MA 02120 USA; 8 Departments of Mechanical and Industrial Engineering, and Bioengineering, Northeastern University, 360 Huntington Ave, SN 257, Boston, MA 02115 USA

**Keywords:** Chiari malformation type I, Integrated longitudinal impedance, Computational fluid dynamics techniques, Displacement encoding with stimulated echoes

## Abstract

**Purpose:**

Chiari malformation type I (CMI) patients have been independently shown to have both increased resistance to cerebrospinal fluid (CSF) flow in the cervical spinal canal and greater cardiac-induced neural tissue motion compared to healthy controls. The goal of this paper is to determine if a relationship exists between CSF flow resistance and brain tissue motion in CMI subjects.

**Methods:**

Computational fluid dynamics (CFD) techniques were employed to compute integrated longitudinal impedance (ILI) as a measure of unsteady resistance to CSF flow in the cervical spinal canal in thirty-two CMI subjects and eighteen healthy controls. Neural tissue motion during the cardiac cycle was assessed using displacement encoding with stimulated echoes (DENSE) magnetic resonance imaging (MRI) technique.

**Results:**

The results demonstrate a positive correlation between resistance to CSF flow and the maximum displacement of the cerebellum for CMI subjects (*r* = 0.75, *p* = 6.77 × 10^−10^) but not for healthy controls. No correlation was found between CSF flow resistance and maximum displacement in the brainstem for CMI or healthy subjects. The magnitude of resistance to CSF flow and maximum cardiac-induced brain tissue motion were not statistically different for CMI subjects with and without the presence of five CMI symptoms: imbalance, vertigo, swallowing difficulties, nausea or vomiting, and hoarseness.

**Conclusion:**

This study establishes a relationship between CSF flow resistance in the cervical spinal canal and cardiac-induced brain tissue motion in the cerebellum for CMI subjects. Further research is necessary to understand the importance of resistance and brain tissue motion in the symptomatology of CMI.

## Introduction

Chiari malformation type I (CMI) is a disorder anatomically characterized by the descent of the cerebellar tonsils greater than 5 mm below the foramen magnum (FM) [1, 2]. Subjects with CMI often suffer from a number of symptoms including, headaches, neck pain, swallowing difficulty, hoarseness, unsteady gait, poor hand coordination, numbness, tingling, dizziness, impaired cognitive ability or dysfunction, depression, and anxiety [3–8]. CMI has traditionally been diagnosed using the cerebellar tonsillar position (CTP); however, large clinical studies conducted retrospectively have indicated that there is not always a relationship between CTP and the severity of symptoms [9, 10]. CMI subjects with large CTP may exhibit only minor neurological symptoms and vice versa [11]. Moreover, a detailed questionnaire was used by Bolognese et al. [12] in 2019 to collect the opinions of 63 globally renowned CMI experts from four continents, all of whom had a combined experience of performing over 15,000 CMI surgeries. The majority of the participants (more than 85%) disagreed with the 5 mm definition as a valid criterion for diagnosing CMI. Research has therefore concentrated on studying structures other than the cerebellar tonsils that may be altered in CMI subjects [13–22].

Integrated longitudinal impedance (ILI) is a hydrodynamic parameter that quantifies unsteady resistance to cerebrospinal fluid (CSF) flow in the spinal canal [13–16]. Researchers have utilized ILI to determine the flow resistance to unsteady fluid motion at a given frequency inside conduits, such as veins, and the spinal canal [23–25]. Shaffer et al. [14] reported that the ILI for CMI subjects was more than twice that of healthy controls. Also, Ibrahimy et al. [16] compared the CSF flow resistance for CMI subjects with cough-associated headaches (CAH) and non-cough-associated headaches (non-CAH) and showed that the value of ILI for CAHs was 2.7 times greater than that of non-CAHs subjects (776 and 285 dyn/cm^5^, respectively).

Previous studies using different magnetic resonance (MR) sequences, including phase-contrast MR (PC-MR) method [17, 20, 21], balanced fast-field echo (bFFE) cine images [18], two-dimensional fast imaging using steady-state acquisition (2D SSFP) [19], and displacement encoding with stimulated echoes (DENSE) MR images [22], have shown that cardiac-induced brain tissue motion is greater in CMI subjects compared to that of healthy controls, especially near the craniocervical junction (CVJ) [17–22].

DENSE-MRI can quantify neural brain tissue displacement in the midsagittal plane. The advantage of DENSE-MRI over PC-MRI is that it encodes displacement directly into the phase of the image instead of requiring error-accumulating path integrations [26]. Additionally, DENSE-MRI can encode displacements that are smaller than the spatial resolution of the acquired images (peak displacements of less than 200 µm) [27–32]. Moreover, Nwotchouang et al. [33] quantified the accuracy of DENSE using a cyclical motion-induced tissue phantom and found that DENSE-MRI demonstrated to be accurate (error ~  < 13 microns) in measuring displacement in cyclical motion using a tissue phantom, which resembles motion measured in human brain tissue induced by cardiac pulsation.

Since CSF flow resistance and cardiac-induced brain tissue motion both have been found to be elevated in CMI subjects, we *hypothesized* a positive correlation existed between resistance to CSF motion and cardiac-induced brain tissue motion. We examined 32 CMI subjects and 18 healthy controls to test this hypothesis. Resistance to CSF motion as quantified by ILI was computed based on computational fluid dynamics (CFD) simulations of the CSF motion based on the subarachnoid space geometry obtained from head/neck MRI. Brain tissue displacement in the brainstem and cerebellum was measured using DENSE MRI. In addition, we examined whether the resistance to CSF motion or brain tissue motion was different for CMI subjects with and without five common symptoms.

## Material and methods

### Study participants

The relevant institutional review boards (IRB) of Emory University approved this study. The MRI scans were acquired at Emory University under an IRB-approved research protocol. Written informed consent was provided by all participants (IRB#8711).

Thirty-two CMI subjects and eighteen healthy controls were analyzed by determining ILI at the cervical spinal canal and displacement of the brainstem/cerebellum. All CMI subjects had a cerebellar tonsillar position (CTP) greater than 5 mm as measured in midsagittal T2-weighted MRI anatomical images. The CMI subjects underwent a survey to identify prevalent clinical symptoms. Out of the 32 individuals with CMI, 31 of them had their clinical information analyzed based on five specific symptoms. The symptoms are imbalance, vertigo, swallowing difficulties, nausea or vomiting, and hoarseness. Also, a total of 25 CMI subjects completed the McGill Pain and Neck Pain Disability Index Questionnaires.

### Model construction for CFD studies

#### Unsteady resistance

**Fig. 1:**
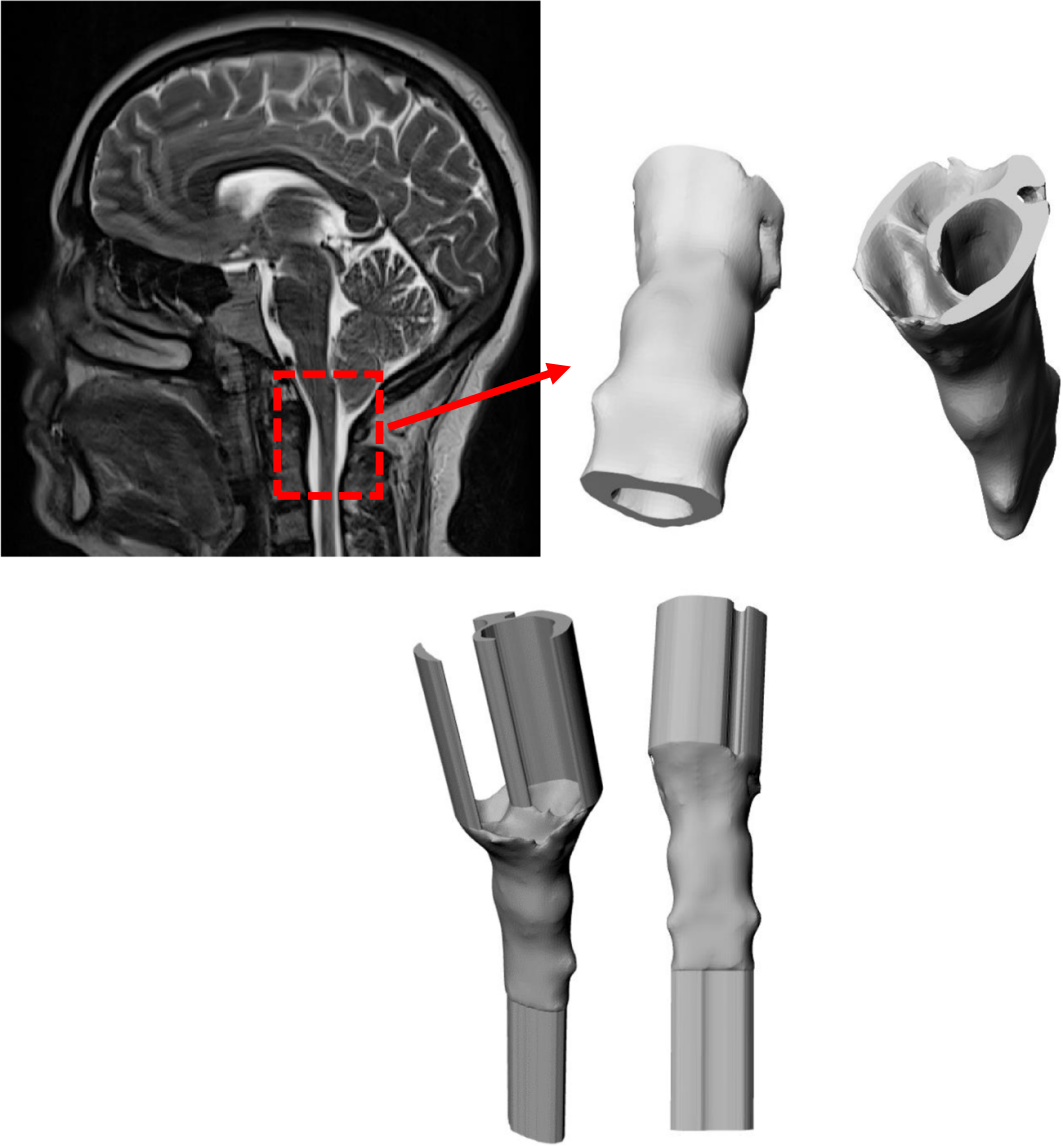
Reconstructed geometry of the upper cervical subarachnoid space

Axial T2-weighted images with an axial slice thickness of 0.8–1.1 mm and pixel spacing of 0.78 × 0.78 – 1.15 × 1.15 mm was used to segment the spinal subarachnoid space to create a three-dimensional (3D) model of this volume in each subject. ITK-SNAP open-source software (ITK-SNAP 4.0 developed by the University of Pennsylvania in collaboration with the University of Utah) was employed to segment the annulus-shaped 3D geometry in the axial plane that extends from FM to an axial plane 60 mm caudal to the FM (near C3 or C4) using semi-automatic active contour (snake) segmentation tool [34]. Pixel artifacts were removed with the Laplacian smoothing algorithm option in the software (MESHLAB, Pisa, Italy). To achieve a nearly fully developed pulsatile flow in the model geometry, 40 mm inlet and outlet extensions were added to the 3D geometry (Autodesk Maya, San Rafael, CA) (Fig. 1).

A computation mesh of the 3D model was created using ANSYS ICEM CFD (Ansys, Inc., Canonsburg, PA). Two types of Tetra/Mixed mesh methods were used to make an unstructured tetrahedral mesh: the Octree and the Delaunay methods. Depending on the shape and size of the 3D model, the mesh ranged between 0.4 and 0.9 million tetrahedral elements. A time-dependent velocity inlet boundary condition was specified at the inlet and a zero-pressure boundary condition was assumed at the flow outlet. No slip (zero velocity) boundary conditions were specified at the walls. The flow waveform input condition is the same for all geometries as ILI is not dependent on the frequency, magnitude, or shape of the CSF waveform [13, 16, 23].

The inlet peak Reynolds number was computed based on the peak flow rate (*Q*), CSF density $(\uprho )$, CSF viscosity $(\upmu )$, and hydraulic diameter (${D}_{H}$) of the spinal canal at the inlet using Eq. 1 and 2:

1 $$Re=\frac{4\rho Q}{\pi \mu {D}_{H}}$$

2 $${D}_{H}=\frac{4A}{P}$$

where *A* is the cross-sectional area of the conduit, and *P* is the wetted perimeter.

CSF was modeled as water at 37 °C with a density of 1.0 g/cm^3^ and a viscosity of 0.01 poise because of its similar properties as those of water at body temperature [35].

**Fig. 2:**
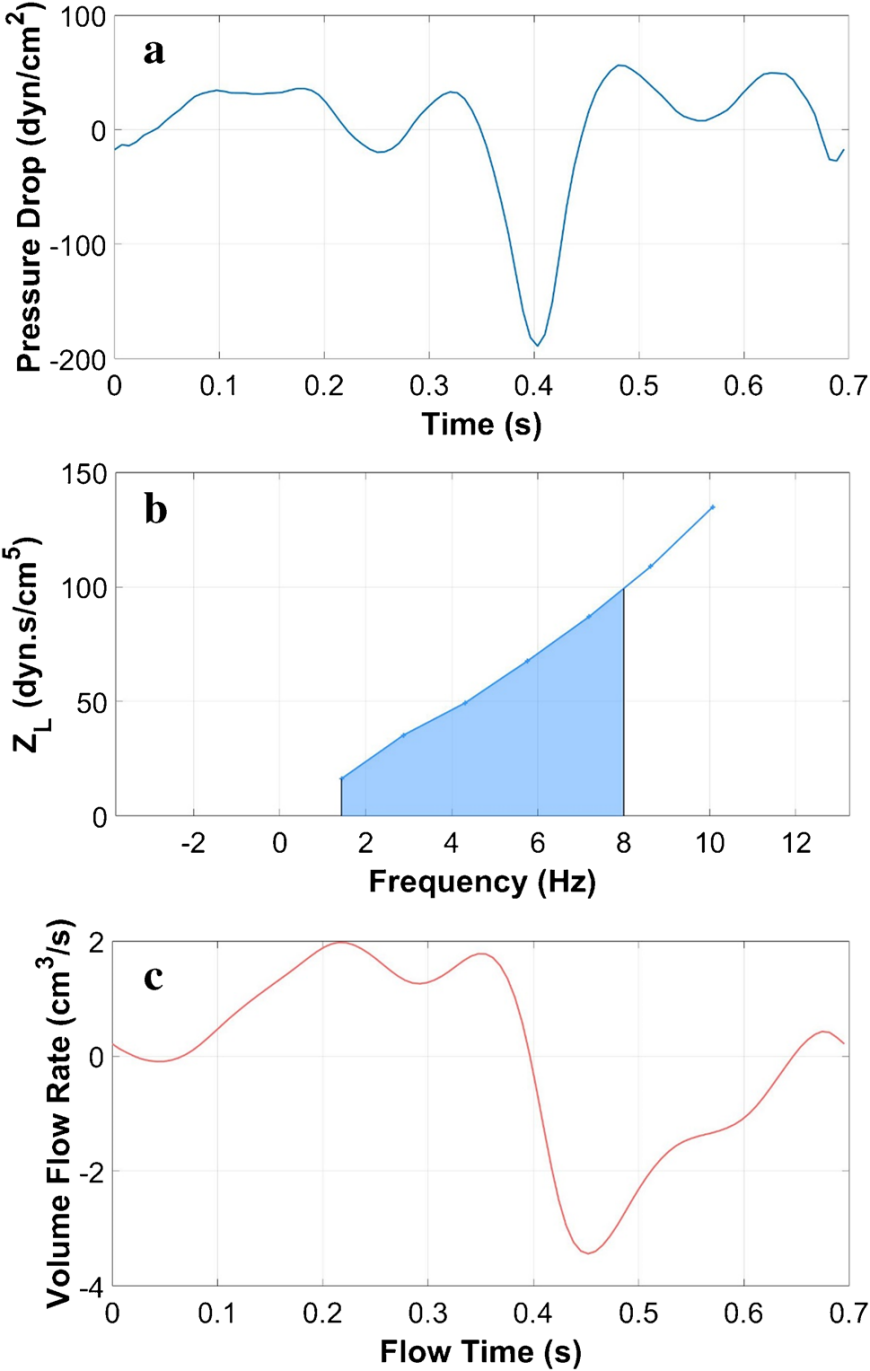
CFD simulation results for one subject: **a** Pressure drop between the FM and a location 25 mm below the FM, **b** ILI (center), and **c** volume flow rate (bottom)

CFD simulations were performed using a finite volume solver (ANSYS FLUENT, Ansys, Inc., Canonsburg, PA). This software applied a discretized form of the Navier–Stokes equation using an upwind second-order scheme and Green-Gauss node-based spatial discretization, and SIMPLE pressure velocity coupling. Convergence criteria were set to 10^−5^ as the minimum residual for continuity and the *x*, *y*, and *z* velocities. Simulations were run with a maximum of 80 iterations to achieve convergence and 100 time steps per cardiac cycle (6.953 ms, heart rate = 86.3 bpm) over three cycles. At every time step, the pressure at each slice location was spatially averaged and assessed. The third simulation cycle data were used to calculate LI in order to avoid startup effects. To calculate LI (*Z*_*L*_), the Fourier transform coefficients of the pressure drop time trace between the two locations (the FM and 25 mm caudal to the FM) were divided by the Fourier transform coefficients of the volume flow rate time trace based on the average pressure across the axial plane, $\Delta P\left(t\right)$ (Fig. 2).

3 $${Z}_{L}=\frac{\mathcal{F}(\Delta P\left(t\right))}{\mathcal{F}(Q\left(t\right))}$$

ILI was computed as the integral of LI for each harmonic from 1 to 8 Hz.

#### Imaging protocol to calculate displacement

**Fig. 3:**
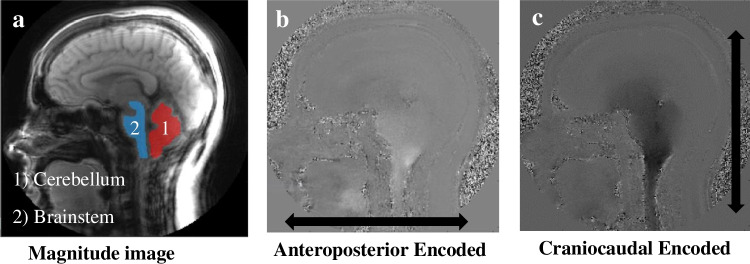
Example midsagittal DENSE MR image set: **a** magnitude image, **b** phase image with displacement encoded in the anterior–posterior direction, and **c** phase image with displacement encoded in the cranial-caudal direction. Black arrows show the displacement encoding direction

A Siemens Healthcare PrismaFit 3 T MRI scanner (Siemens Medical, Erlangen, Germany) equipped with a 20-channel head coil acquired sagittal T1 and T2 weighted images of the brain for all subjects. Peripheral pulse unit gating was used to acquire midsagittal spiral cine DENSE scans with encoding for displacement in the anterior–posterior direction and the cranial-caudal direction (Fig. 3) [36]. The DENSE acquisition was carried out using imaging parameters listed in Table 1.

**Table 1:** Imaging protocol and parameters

Parameter	Value
MRI scanner	Siemens PrismaFit 3 T
Head coil	20 channel
Weighted images	Sagittal T1 and T2
Displacement scan	Midsagittal spiral cine DENSE
Displacement directions	Anterior–posterior, cranial-caudal
Flip angle	15°
Temporal resolution/frame	34 ms
Encoding frequency	0.6 cycles/mm
Spiral interleaves/heartbeat	2
Total spiral interleaves	192
Field of view	256 × 256
Reconstruction matrix	256 × 256
Pixel size	0.86–0.94 × 0.86–0.94 mm
Slice thickness	8 mm

#### Post-processing

DENSE scans were post-processed using a custom program developed using MATLAB (MathWorks, Natick, MA), and the procedures for obtaining displacement were the same as those described by Pahlavian et al. [30]. The brain regions were identified using magnitude images (Fig. 3a). Anterior–posterior and cranial-caudal displacements were determined based on phase images (Fig. 3b, c). Displacements were computed after phase unwrapping (if needed), and noise filtering, in selected brain regions (cerebellum and brainstem shown in Fig. 3a. Noise filtering was applied using a finite impulse response filter to the displacement measurement using a normalized cutoff frequency and filter order of 0.15 and 16, respectively (*filter2* function in MATLAB 2022a) [30]. To create pixel-wise Eulerian displacement maps for the brain regions, an interactive paintbrush tool in MATLAB was used to segment the brain regions in the magnitude and phase images. To calculate the maximum spatial displacement in the brainstem and cerebellum, a circular region of interest (ROI) of approximately 30 mm^2^ was identified at the location that exhibits the largest displacement during the cardiac cycle. The largest value of cardiac-induced displacement during the cardiac cycle in this circular ROI will henceforth be referred to as “displacement” in the text.

#### Symptom assessment

CMI subjects often suffer from pain and neck-pain related disability symptoms. In order to explore the correlation between the biomechanical parameters (ILI, displacement at the cerebellum, and brainstem) and these symptoms, twenty-five out of thirty-two CMI subjects were administered two standardized clinical self-report questionnaires: the ShortForm McGill Pain Questionnaire (MPQ) [37], and the Neck Pain Disability Index Questionnaire (DIQ) [38].

#### Statistical analysis

Correlations were conducted between ILI and displacement in both cerebellum and brainstem for CMI and healthy subjects. Linear regression analysis was performed for each of the four cases (non-zero constant, 95% confidence, Microsoft Excel). Correlation results were considered to be statistically significant for *p*-values less than 0.05. ILI and displacement at the cerebellum and brainstem for CMI subjects were compared between subjects with and without a specific clinical symptom using an unpaired Student’s *t*-test with unequal variances (Microsoft Excel). To test for normality in the data, the Shapiro–Wilk test was utilized. Clinical information about five symptoms was used for 31 of the 32 CMI subjects. Differences were considered statistically significant for *p*-values less than 0.05.

## Results

**Fig. 4:**
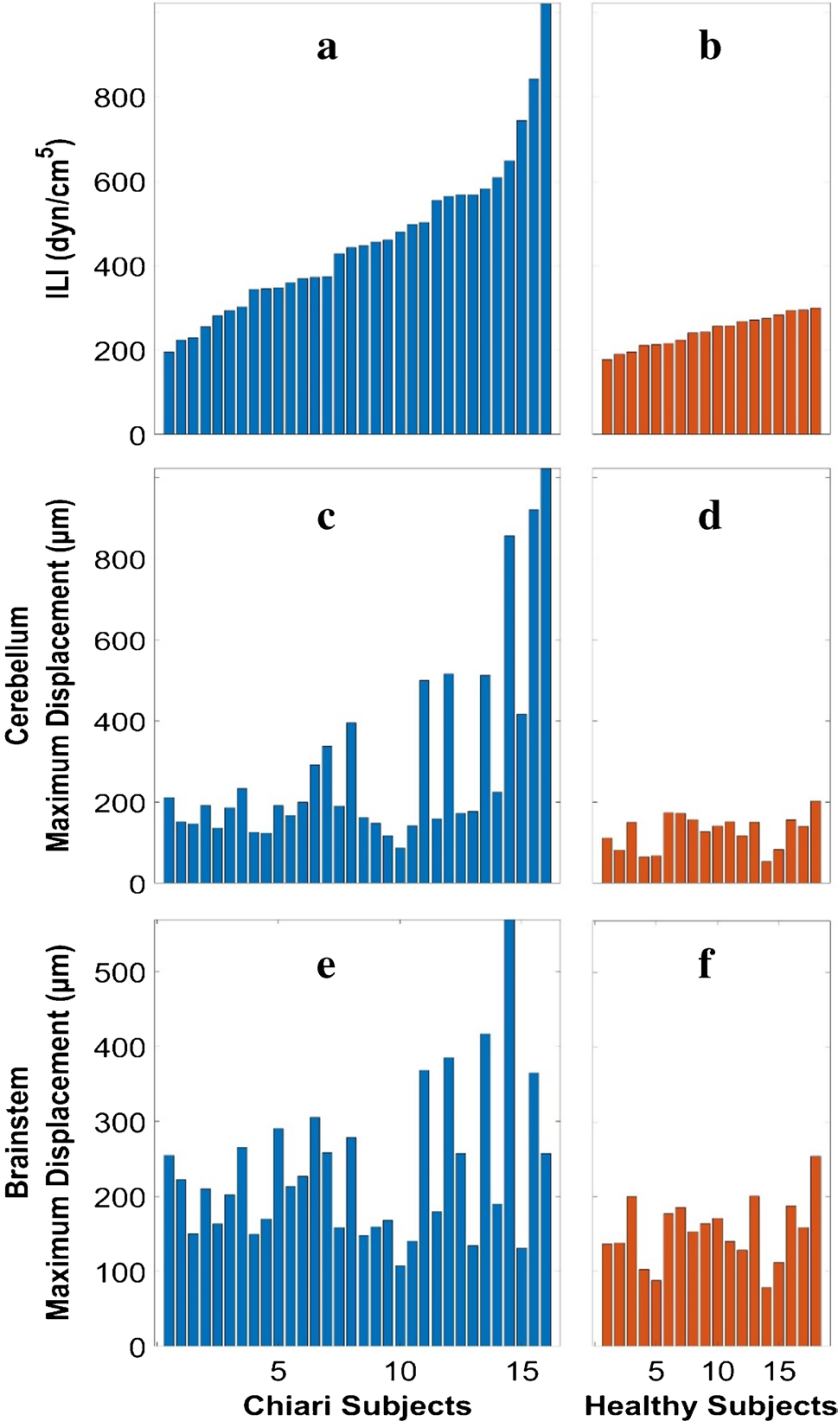
ILI values in order of magnitude for CMI subjects (**a**) and healthy controls (**b**). Brain tissue displacement at the cerebellum (**c**, **d**) and brainstem (**e**, **f**) for CMI subjects and healthy controls in the same order as in (**a**, **b**)

**Fig. 5:**
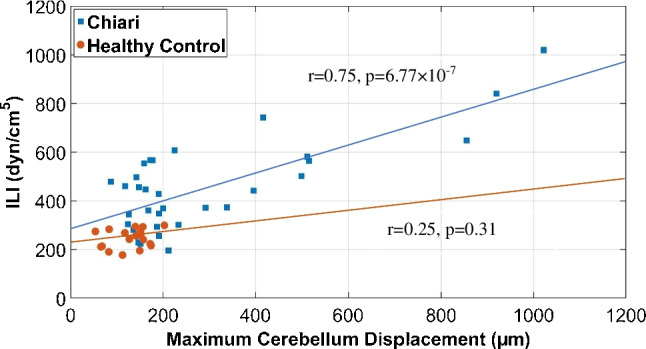
ILI versus displacement at the cerebellum for Chiari subjects and healthy controls

**Fig. 6:**
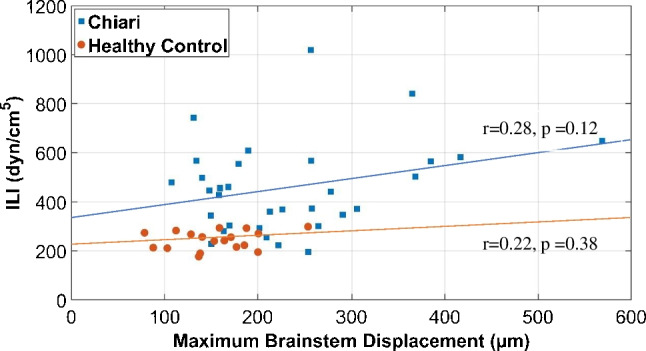
ILI versus displacement at the brainstem for Chiari subjects and healthy controls

Significantly higher ILI was found in CMI subjects compared to healthy controls (485 ± 184 dyn/cm^5^ for CMI vs. 244 ± 38 dyn/cm^5^ for healthy; *p* = 1.3 × 10^−7^). Significantly higher displacement was found in CMI subjects compared to healthy controls at both the cerebellum (294 ± 240 µm for CMI vs. 128 ± 42 µm for healthy; *p* = 0.0003) and brainstem (234 ± 101 µm for CMI vs. 154 ± 44 µm for healthy; *p* = 0.000175) as shown in Fig. 4. For CMI subjects, a statistically significant correlation was observed between the ILI and displacement in the cerebellum (*r* = 0.75, *p* = 6.8 × 10^−7^; Fig. 5) but not for the brainstem (*r* = 0.25, *p* = 0.31; Fig. 6). For healthy controls, no statistically significant correlation was observed between ILI and displacement for either brain region as shown in Figs. 5 and 6.

No statistically significant differences were observed for any of the three biomechanical parameters examined (ILI, displacement at the cerebellum, and brainstem) when comparing CMI subjects with and without the five symptoms examined. The frequency of each symptom was imbalance (12/31), vertigo (19/31), swallowing difficulties (16/31), nausea or vomiting (16/31), and hoarseness (13/31).

The mean (standard deviation) of the pain indices were 106.9 (51.5) for the MPQ and 50.9 (17.9) for the DIQ, respectively. The results showed that there was no relationship between each of the biomechanical parameters (ILI, displacement at the cerebellum, and brainstem) and the mean of the pain indices.

## Discussion

CSF flow resistance in the spinal canal and cardiac-induced brain tissue displacement of the cerebellum and brainstem were examined for 32 CMI subjects and 18 healthy controls. The results showed a significant correlation between CSF flow resistance and cerebellar displacement for CMI subjects but not for healthy controls. No correlation was found between CSF flow resistance and brainstem displacement in CMI subjects or healthy controls. No differences were observed between the mean values of CSF flow resistance, cerebellar displacement, and brainstem displacement in CMI subjects with and without five common CMI symptoms.

Previous studies have measured ILI in CMI subjects and healthy controls and showed significantly higher values of ILI in CMI subjects compared to healthy controls [13, 14]. Shaffer et al. [14] showed that the mean ILI values for CMI patients and healthy controls were 551 and 220 dyn/cm^5^, respectively, which is similar to the results of the present study with mean ILI value for CMI subjects and healthy controls of 485 and 244 dyn/cm^5^, respectively. Previous work has shown ILI decreases after posterior fossa decompression surgery [13] which is similar to the decrease observed for brain tissue motion after surgery [39].

Brain tissue motion has been shown to be significantly higher in CMI subjects compared to healthy controls near the cervicomedullary junction using various MR methodologies [17, 19–22]. For example, Alperin et al. [17] utilized PC-MRI method to compare the maximum displacement of the cord near C2 level of thirty-four CMI subjects and seventeen healthy controls and found that the maximum displacement was 390 and 330 µm for the CMI subjects and healthy controls, respectively. Lawrence et al. [20] compared spinal cord motion at the level of FM in CMI subjects before and after surgery and healthy controls using PC-MRI and demonstrated that spinal cord motion in pre-surgical subjects was 231% greater than that of healthy controls. Additionally, their study demonstrated a significant decrease in spinal cord displacement after the surgery. Wolpert et al. [21] evaluated the motion of the cerebellar tonsils, medullae, and upper cervical cord in nine CMI subjects and eight healthy controls using PC-MRI and reported approximately 10 times greater cerebellar tonsils tissue velocity in CMI subjects compared to healthy controls. Leung et al. [18] used bFFE cine images and reported 33% greater cerebellar tonsillar displacement in CMI subjects compared to healthy controls. Nwotchouang et al. [22] reported significantly higher motion in the cerebellum and brainstem of CMI subjects compared with the healthy controls. The results presented herein are similar to that of Nwotchouang et al. [22] and Eppelheimer et al. [39]. This could be because all measurements were done with the same methodology (DENSE). In addition, there is partial overlap between the subjects presented herein and those of Nwotchouang et al. [22]. Specifically, out of the 50 subjects in our dataset, 38 of them overlapped with the subjects included in Nwotchouang’s study. The results of the two studies were comparable with maximum displacement in the cerebellum found to be 369 and 294 µm for CMI subjects and 120 and 128 µm for healthy controls for Nwotchouang et al. [22] and the present study, respectively. Similarly, maximum displacement in the brainstem was 300 and 234 µm for CM subjects and 148 and 154 µm for healthy controls for Nwotchouang et al. [22] and the present study, respectively. However, when comparing displacement to other techniques, the magnitudes were considerably different. At the level of the foreman magnum, Lawrence et al. [20] reported spinal cord motion of 530 µm and 230 µm in the CMI and control groups, respectively, which is considerably larger than the brainstem motion herein. Alperin et al. [17] evaluated the maximum cord displacement near the C2 level and found 390 µm and 330 µm for CMI and control groups, respectively. This difference is smaller than that reported by Lawrence et al. [20] and our results in the brainstem. Cousins et al. [19] measured cardiac-induced brain tissue motion for cerebellar tonsils using 2D FIESTA and found an average cerebellar tonsillar displacement of 570 and 430 µm which is much larger than the values obtained in this study. Differences in measurement location, processing method, and MR protocol may be the cause of the wide variation in cardiac inducted tissue motion.

The decrease in CSF space causes pressure dissociation near the FM, resulting in large tissue motion [20]. In CMI subjects with increased neural tissue velocity, Wolpert et al. [21] proposed that the increased tissue velocity is partly due to a systolic pulse wave and limited CSF cushioning, forcing tissue into the spinal canal, and partly due to the Bernoulli effect induced by crowding near the cerebellar tonsils. In short, the altered geometry of CMI subjects results in larger resistance to CSF motion which, in turn, creates larger pressure gradients during the cardiac cycle which causes greater cardiac-induced brain tissue motion. The results of the present study agrees with this concept as a significant correlation (*r* = 0.75) between ILI and brain tissue displacement in CMI subjects for the cerebellum was observed. However, no correlation was observed in healthy controls for either the brainstem or cerebellum. There may be a threshold for the resistance such that subjects with less crowding do not demonstrate this relationship. To test this idea, we conducted linear regression on subsets of the CMI subjects with minimum values of tissue motion of 150, 200, and 250 µm and found stronger correlation with larger ILI cutoff values (Table 2). For example, ILI and brain tissue motion were significantly correlated with an *r* = 0.86 for a subset of patients with tissue motion larger than 250 µm. It is unclear why there are many CMI cases (*n* = 20) with elevated resistance but tissue motion similar to that of healthy controls. For subjects with tissue motion less than 200 µm, the mean ILI for CMI and healthy controls was 393 and 241 dyn/cm^5^, respectively. It is not clear why this correlation was not present in the brainstem although it is worth noting that the increase in tissue motion for CMI patients was much greater for the cerebellum compared to the brainstem (i.e., crowding impacted the brainstem less than the cerebellum). Compliance of the subarachnoid space has been hypothesized to also play a role in CSF hydrodynamics [40]. The variation in compliance between subjects may diminish the correlation between displacement and ILI. Finally, Chiari subjects with relatively low ILI may have symptoms due to something other than crowding of the cervicomedullary junction such as ventral compression, Ehlers-Danlos syndrome, and pseudotumor cerebri.

**Table 2:** Correlation between ILI and displacement at the cerebellum for CMI cases

Cerebellum displacement cutoff	*p*-value	*r*
All cases	6.77 × 10^−7^	0.75
> 150 µm	9.94 × 10^−6^	0.77
> 200 µm	4.73 × 10^−4^	0.82
> 250 µm	1.16 × 10^−3^	0.86

The lack of a relationship between the biomechanical measures and the five different CMI symptoms raises a question as to the importance of ILI and brain tissue motion in the prediction of CMI symptomology. Many of the symptoms examined are brainstem related while the correlation between ILI and displacement was for the cerebellum. Previous research has shown that ILI is predictive of cough-associated headaches for CMI subjects [16]. The brain tissue motion is thought to be associated with nerve damage; however, the level of motion and tissue strain generated by cardiac-induced motion may be insufficient to cause significant damage [41]. Further research should examine the biomechanical parameters in more dynamic states such as coughing or the Valsalva maneuver. These results contribute to the growing body of research on the complex relationship between Chiari malformation, pain, and biomechanical factors [3–7]. Future research could explore other factors that may contribute to pain and discomfort in these patients, such as psychological and social factors, to better understand the nature of the pain experienced by Chiari patients and develop effective treatment strategies.

## Limitations

There were certain limitations associated with this research. This study measured the brain tissue displacements in the sagittal plane in the craniocaudal and posterior-inferior directions. This measurement provides no information about tissue displacement in the lateral direction and previous work has shown that lateral displacement is present but its contribution to the total displacement is small [30]. There is a potential bias in the manual segmentation of the brainstem and cerebellum. A two-to-three-voxel-wide area was excluded from the analysis to reduce the possibility of including unwanted structures in the brain region outline. There is also potential bias in the manual segmentation of the CSF space as part of the methodology for ILI computation since pressure drop is strongly related to lumen diameter. The simulation to compute ILI was conducted assuming a rigid geometry (no tissue motion) based on the average spinal canal geometry during the cardiac cycle. Pahlavian et al. [15] showed ILI magnitude to be 19% greater using a moving tissue model compared to a static model. Thus, the accuracy of our ILI magnitude will be worse for subjects with large motion compared to those with smaller tissue motion.

## Conclusion

The present study showed a significant correlation between CSF flow resistance and brain tissue motion in the cerebellum for CMI subjects. This correlation suggests a potential relationship between these two factors. It can be hypothesized that increased CSF flow resistance might contribute to the displacement of the cerebellum in CMI subjects. Therefore, measuring CSF flow resistance could potentially serve as an additional diagnostic tool in conjunction with existing imaging techniques to aid in the identification and diagnosis of CMI subjects in clinical practice. This result provides evidence for theories that relate increased CSF flow resistance to greater brain tissue motion. This is particularly true for CMI cases with greater crowding. The exact mechanisms underlying the correlation between CSF flow resistance and brain tissue motion in CMI patients are not fully understood, and more research is needed to elucidate these relationships.
